# Marine Protein Sources and Lipid Levels in Microdiets Affect the Survival and Health of Whiteleg Shrimp (*Penaeus vannamei*) Post-Larvae in Clear-Water Recirculating Aquaculture Systems

**DOI:** 10.3390/ani16152413

**Published:** 2026-08-05

**Authors:** André Barreto, Diogo Peixoto, Carlos Fajardo, Sílvia Pires, Wilson Pinto, Rui J. M. Rocha, Luís E. C. Conceição, Benjamín Costas

**Affiliations:** 1 Riasearch Lda, Cais da Ribeira de Pardelhas 21, 3870-168 Murtosa, Portugal; silviapires1@ua.pt (S.P.); ruirocha@riasearch.pt (R.J.M.R.); 2 Centro Interdisciplinar de Investigação Marinha e Ambiental (CIIMAR), 4450-208 Matosinhos, Portugal; dpeixoto@ciimar.up.pt (D.P.); cquinones@ciimar.up.pt (C.F.); bcostas@ciimar.up.pt (B.C.); 3 Instituto de Ciências Biomédicas Abel Salazar (ICBAS-UP), Universidade do Porto, 4050-313 Porto, Portugal; 4 Departmento de Biologia, Facultad de Ciencias del Mar y Ambientales, Instituto Universitario de Investigación Marina (INMAR), Campus de Excelencia Internacional del Mar (CEI·MAR), Universidad de Cádiz, 11519 Puerto Real, Spain; 5 Departamento de Biologia & CESAM, Campus Universitário de Santiago, Universidade de Aveiro, 3810-193 Aveiro, Portugal; 6 Sparos Lda, Área Empresarial de Marim, Lote C, 8700-221 Olhão, Portugal; wilsonpinto@sparos.pt (W.P.); luisconceicao@sparos.pt (L.E.C.C.)

**Keywords:** aquaculture nutrition, larviculture, nursery feeds, nutrient utilization, fatty acid composition, antioxidant response, immune response

## Abstract

This study evaluated the effects of different marine protein sources and dietary lipid levels in inert microdiets for whiteleg shrimp (*Penaeus vannamei*) post-larvae (PL) reared in clear-water recirculating aquaculture systems (RAS). Five diets were evaluated: a control diet containing a blend of fish and squid meals, diets based on fish meal, squid meal, or krill meal as the main marine ingredient, and a high-lipid fish meal-based formulation used to assess the effect of increased dietary lipid content. After 21 days, growth performance did not differ significantly among treatments. However, survival and physiological status were significantly affected by diet. PL fed the control and krill-based diets showed higher survival (76.5 and 78.0%, respectively), whereas survival was lowest with the high-lipid fish meal-based diet (41.5%), which also resulted in an increased feed conversion ratio (FCR). Fatty acid composition reflected dietary inputs, with higher levels of n-3 polyunsaturated fatty acids in PL fed the high-lipid diet. Biomarker and gene expression analyses revealed modulation of antioxidant and immune responses in PL fed single-ingredient diets and the high-lipid formulation, suggesting increased physiological stress compared to the control and krill-based treatments. These results show that growth alone is not sufficient to assess diet adequacy. The use of combined marine protein sources or krill meal was associated with higher survival, while increasing dietary lipid content from 14 to 16% did not improve growth performance and was associated with reduced survival.

## Simple Summary

Shrimp farming relies on high shrimp survival and good health during the early life stages, yet selecting the most suitable feeds at this stage remains challenging. This study evaluated the effects of different marine protein sources (fish, squid, or krill) and lipid levels in microdiets on the growth, survival, and health of whiteleg shrimp post-larvae reared in closed water production systems. All diets supported similar growth, indicating that shrimp were able to gain weight regardless of feed formulation. However, clear differences were observed in survival and health condition. Shrimp fed diets based on a combination of fish and squid meal, or krill meal alone, showed higher survival and better overall condition. In contrast, diets based on a single ingredient or with higher lipid levels reduced survival and were associated with signs of physiological stress. These findings demonstrate that growth alone is not sufficient to assess feed adequacy. The use of combined marine ingredients or krill meal, together with moderate lipid levels, can improve shrimp health and survival. This contributes to more efficient and sustainable shrimp production by reducing losses and supporting animal welfare.

## 1. Introduction

Diets for *P. vannamei* post-larvae (PL) produced in clear-water recirculating aquaculture systems (RAS) require high inclusion levels of protein, as inert feeds are the only source of these nutrients in a system devoid of natural or artificial productivity. As determined in [1], a dietary protein level of at least 47% is recommended to ensure maximum growth and survival, with even greater proportions of up to 54% seemingly being beneficial to the antioxidant status of PL and, potentially, other physiological functions.

Beyond the overall protein level, the quality and complexity of dietary protein sources may play an important role during early developmental stages, when digestive capacity is still developing, and the efficiency of nutrient utilization is highly dependent on ingredient characteristics. In fish larvae, it has been well established that the complexity and digestibility of dietary protein strongly influence its utilization, modulating growth, nutrient use, and digestive processes [2,3,4]. Similar trends have been reported in *P. vannamei*, particularly at juvenile stages, where the use of different marine-derived ingredients has been shown to influence feed intake, survival, and physiological condition, thereby highlighting the functional role of ingredient selection beyond crude nutrient levels [5,6,7,8]. These findings suggest that not all protein sources are equally suitable, and that highly digestible marine ingredients or specific ingredient combinations may provide nutritional and physiological benefits over conventional formulations during early development.

Fish meals have long been the primary ingredient in *P. vannamei* aquafeeds due to their high digestibility, balanced amino acid profile, high nutrient bioavailability, micronutrient richness, and strong feed attractability [9,10]. However, evidence from early life stages suggests that fish meal may not always represent the optimal solution, particularly when compared to other marine-derived ingredients such as squid meal and krill meal, which exhibit high digestibility, attractability, and functional properties. These ingredients have shown beneficial effects in juvenile *P. vannamei*, improving feed intake, survival, and health status [6,7,8], supporting their potential as alternatives to conventional feeds. However, their relative suitability as marine protein sources for post-larval diets remains largely unexplored.

Therefore, this study aimed to evaluate how different marine protein sources and dietary lipid levels in inert microdiets influence the growth performance, survival, and physiological status of *P. vannamei* PL reared in clear-water RAS.

## 2. Materials and Methods

### 2.1. Dietary Treatments

Five experimental microdiets were evaluated in triplicate. A control diet (CTRL) was formulated containing 54% protein and 14% lipids, utilizing a blend of fish and squid meals as the main ingredients. Diets FISH, SQUID, and KRILL were formulated to contain the same levels of protein and lipids but with fish, squid, and krill meals, respectively, as the main ingredients. Diet FISHHF had a similar composition to that of FISH but was formulated to contain a higher lipid content (16%) through higher inclusion levels of fish oil, allowing the effects of lipid enrichment to be evaluated while maintaining the same primary marine protein source. The composition of the diets is described in Table 1 and the ingredient formulation in Table 2. All diets were produced at the Sparos Lda facilities (Olhão, Portugal) using low-temperature extursion as the main production process, as follows: powder ingredient mixing according to target formulation using a double-helix mixer; grinding in a micropulverizer hammer mill (SH1, Hosokawa–Alpine, Augsburg, Germany); addition of the oil fraction; humidification and agglomeration through low temperature extrusion (Dominioni Group, Camisano, Italy); drying of the resultant pellets in a convection oven (OP 750-UF, LTE Scientifics, Oldham, United Kingdom) for 4 h at 60 °C, crumbling (Neuero Farm, Melle, Germany) and sieving to the desired size ranges.

### 2.2. Shrimp Rearing and Sampling

*P. vannamei* PL15 (mean wet weight of 13.0 ± 2.0 mg), all originating from the same production batch from Miami Aquaculture (Boynton Beach, FL, USA), were reared for 21 days at RIASEARCH Lda facilities (Murtosa, Portugal). They were randomly distributed among 15 tanks (approximately 50 L each), part of a 3 m^3^ clear-water recirculating system, at a larval density of 250 individuals per tank (shrimp density of 5000 m^−3^). The shrimp were kept under a 12 h light:12 h dark photoperiod and were fed close to ad libitum with automatic feeders that supplied eight meals a day. Feeders were cleaned daily and charged with adjusted feed quantities based on feeding activity and the presence or absence of feed remnants from the previous day, following a standardized feeding management procedure. Feed size was 400–600 µm for the first week and 600–800 µm for the remaining feeding period. Water parameters were monitored daily. Temperature was maintained at 27.6 ± 0.5 °C, dissolved oxygen concentration at 6.6 ± 0.3 mg L^−1^, salinity at 22.7 ± 1.1 g L^−1^, pH at 8.1 ± 0.1, NH_3_ and NO_2_ at 0.0 ± 0.0 mg L^−1^.

At the start of the trial, a total of 120 PL from the initial stock were randomly selected and weighed in groups of 30 individuals for initial wet weight determination. At the end of the experiment, a total of 50 PL were randomly selected per tank and weighed individually. The remaining PL were counted and weighed in groups to determine the final tank biomass and survival. For whole-body composition analysis, a total of 10 g of PL were randomly collected from each tank. Additionally, 23 PL were randomly selected from each tank for oxidative stress, energy reserves, immune parameters, and gene expression analysis by RT-qPCR. These samples were stored at −80 °C for subsequent analysis. PL sampled for molecular biology analysis were kept in RNAlater (Sigma–Aldrich, St. Louis, MO, USA) at a >1:5 volume ratio, at 4 °C for 24 h prior to being stored at −20 °C. PL were fasted for 12 h prior to sampling to minimize gut content and ensure a more standardized metabolic state among individuals.

### 2.3. Oxidative Stress-Related Biomarkers and Cellular Energy Allocation

#### 2.3.1. Sample Preparation

A total of 9 *P. vannamei* PL from each tank sampled at the end of the trial were weighed and homogenized in three groups of 3 individuals by sonication on ice using 2800 μL of ultra pure water. Three aliquots of 300 μL were taken from each sample for the analysis of lipid, carbohydrate, and protein contents. An aliquot for lipid peroxidation (LPO) with butylated hydroxytoluene was reserved prior to centrifugation. The remaining homogenate (~700 μL) was diluted with 700 μL of 0.2 M K-phosphate buffer, pH 7.4, and centrifuged for 20 min at 9000× *g* (4 °C). From the post-mitochondrial supernatant (PMS), aliquots for the measurement of catalase (CAT), glutathione S-transferase (GST), total glutathione (tGSH), and protein content were taken.

#### 2.3.2. Determination of Oxidative Stress Biomarkers

Protein concentration of PMS was determined following the Bradford method [11], adapted from BioRad’s Bradford micro-assay set up in a 96-well flat-bottom plate, with a standard of bovine γ-globulin.

CAT activity levels were determined by measuring the decrease in hydrogen peroxide (H_2_O_2,_ 30%, Sigma–Aldrich, St. Louis, MO, USA) concentration, as described by [12]. The reaction mixture was composed of K-phosphate buffer (0.05 M, pH 7.0) and H_2_O_2_ (30%) as substrate. A total of 10 µL of homogenate sample was added to the reaction mixture, resulting in a total volume of 300 µL. Absorbance was read at 240 nm in UV microplates for 2 min (1 reading every 15 s), and results were expressed as μmol min^−1^ mg^−1^ protein.

Endogenous LPO was assessed by measuring thiobarbituric acid-reactive substances (TBARS), while preventing artefactual lipid oxidation by adding butylhydroxytoluene (4%; Sigma–Aldrich, St. Louis, MO, USA) [13]. Homogenate samples were incubated for 60 min at 100 °C with 100 µL of 100% trichloroacetic acid solution and 1 mL of 2-thiobarbituric acid 0.73% (Sigma–Aldrich, St. Louis, MO, USA), trizma hydrochloride (Sigma–Aldrich, St. Louis, MO, USA), and diethylenetriaminepentaacetic acid (Fluka, Buchs, Switzerland) solution. Afterward, the samples were centrifuged for 5 min at 11,500 × rpm, and 200 µL of the supernatant were added to the microplate wells. Absorbance was read at 535 nm, and results were expressed as nmol TBARS g^−1^ tissue.

GST activity was determined by following the conjugation of reduced glutathione (GSH) with 1-chloro-2,4-dinitrobenzene (CDNB) as described by [14]. Absorbance was read at 340 nm, and results were expressed as nmol min^−1^ mg^−1^ protein.

Levels of tGSH in PL homogenate samples were measured based on the oxidation of glutathione by 5,5′-dithiobis-(2-nitrobenzoic acid) (DTNB; Sigma–Aldrich, St. Louis, MO, USA) as described by [15]. Samples were diluted in K-phosphate buffer (0.1 M, pH 7.4) to obtain 0.7 mg mL^−1^ of protein. Thereafter, 50 µL of each diluted sample were added to microplate wells, followed by the addition of 250 µL of a reaction solution, composed of DTNB, K-phosphate buffer (0.1 M, pH 7.4), NADPH (ß-nicotanimide adenine dinucleotide 2′-phosphate reduced tetrasodium salt; Alpha Aesar, Haverhill, MA, USA) and glutathione reductase (Sigma–Aldrich, St. Louis, MO, USA). Absorbance was read at 412 nm for 3 min (1 reading every 20 s), and results were expressed as nmol min^−1^ mg^−1^ protein. All biomarker determinations were performed with the Multiskan Spectrum microplate reader (Thermo Fisher Scientific, Waltham, MA, USA).

#### 2.3.3. Determination of Cellular Energy Allocation

Energy available (EA, assessed as the sum of lipids, carbohydrates, and proteins) and energy consumption (EC, as electron transport system—ETS activity) were determined by following the methods described by [16] adapted for a microplate format [17]. All the values obtained were adjusted to PL weight. The final CEA value was calculated as: CEA = EA/EC [18].

Total lipid contents were determined by adding chloroform (119.38 M; ACS spectrophotometric grade, ≥99.8%), methanol (32.04 M; ACS reagent, ≥99.8%), and Milli-Q water in a 2:2:1 proportion to 300 µL of the homogenized samples. The samples were then centrifuged (1000× *g*, 5 min, 25 °C) and the organic phase of each sample was transferred to clean glass tubes. A total of 500 μL of sulfuric acid (H_2_SO_4_) was added, and the samples were incubated for 15 min at 200 °C. Afterward, 1500 μL of ultra pure water was added to each sample, and absorbance was measured at 375 nm. Tripalmitin was used as a calibration curve. Carbohydrates and proteins were measured in the same aliquot using 100 μL of 15% trichloroacetic acid (TCA), followed by incubation for 10 min at −20 °C and then centrifugation at 1000× *g* for 10 min at 4 °C, to separate both components. Afterward, 200 μL of 5% phenol and 800 μL of H_2_SO_4_ were added to the supernatant to measure carbohydrate content using glucose as a standard, and the absorbance was read at 492 nm. The pellet obtained from the previous centrifugation was resuspended in 500 µL of sodium hydroxide (NaOH), incubated (30 min at 60 °C), and neutralized with 280 µL of hydrochloric acid (HCl). It was subsequently used for protein quantification using bovine serum albumin as a standard curve, according to the method described by [11], by adding Bradford reagent to the microplates, with the absorbance measured at 592 nm. Fractions of energy available were converted into energetic equivalent values using the corresponding energy of combustion: 39,500 mJ g^−1^ lipids, 17,500 mJ g^−1^ glycogen, and 24,000 mJ g^−1^ protein [16]. The final value of EA was calculated as the sum of lipid, carbohydrate, and protein content in mJ mg^−1^ tissue.

ETS activity was measured in 300 µL of the homogenized samples, to which 150 μL of homogenization buffer (0.3 M Tris base; 0.45% (*w*/*v*) polyvinylpyrrolidone; 459 µM magnesium sulphate (MgSO_4_); 0.6% (*v*/*v*) Triton X-100 at a pH of 8.5) was added and then centrifuged (1000× *g*, 10 min, 4 °C). Consequently, 50 μL of supernatant from each replicate was transferred to a microplate, and 150 μL of buffered solution B (solution A (0.13 M Tris base; 0.27% (*v*/*v*) Triton X-100); 1.7 mM reduced nicotinamide adenine dinucleotide (NADH); 274 μM NADPH); and 100 μL of INT solution (p-iodonitrotetrazolium; 8 mM) were added to start the reaction. Absorbance was measured at 490 nm for 3 min. The cellular oxygen consumption rate was calculated based on the stoichiometric relationship of 2 μmol of formazan formed to 1 μmol of oxygen consumed. The quantity of oxygen consumed was determined by the Lambert–Beer formula: A = ε × l × c (A = absorbance; ε for INT-formazan = 15,900/M.cm; l = 0.9; c = oxygen consumed). The EC value was obtained by converting oxygen consumption to energetic values using the specific oxyenthalpic equivalent for an average lipid, carbohydrate, and protein mixture of 480 kJ mol O_2_^−1^.

### 2.4. Immune Condition Parameters

#### 2.4.1. Sample Preparation

A total of 9 *P. vannamei* PL from each tank sampled at the end of the trial were weighed and homogenized in three groups of 3 individuals, as described by [19]. Potassium phosphate buffer (0.1 M) was added to each group in a 1:10 (*w*/*v*) proportion, followed by homogenization using a high-performance dispersing instrument (SilentCrusher M, Heidolph Instruments, Schwabach, Germany). After centrifugation (5500 rpm for 20 min), the sample supernatant was collected and distributed into separate aliquots for lysozyme and pro-phenoloxidase activities.

#### 2.4.2. Determination of Immune Parameters

Lysozyme activity was measured using a turbidimetric assay, as described by [20]. Briefly, a solution of *Micrococcus lysodeikticus* (0.25 mg mL^−1^, 0.05 M sodium phosphate buffer, pH 6.2) was prepared, and 40 μL of homogenized samples and 130 μL of this suspension were added to a microplate, resulting in a final volume of 170 μL. The reaction was carried out at 25 °C, and absorbance at 450 nm was measured after 0.5 and 30 min using a Synergy HT microplate reader (BioTek Instruments, Winooski, VT, USA). Lyophilized hen egg white lysozyme (Sigma–Aldrich, St. Louis, MO, USA) was diluted in sodium phosphate buffer (0.05 M, pH 6.2) and used to develop a standard curve. The amount of lysozyme in the sample was calculated using a standard curve. Lysozyme activity was expressed as µg mg^−1^ protein.

Pro-phenoloxidase activity was measured spectrophotometrically using L-3,4- dihydroxyphenylalanine (L-DOPA) as substrate, and trypsin (Sigma–Aldrich, St. Louis, MO, USA) as the activator, based on the method described by [21]. Homogenate samples of 50 μL were diluted in 100 μL of trypsin solution (0.1% in cacodylate solution) in a 96-well microplate and incubated for 30 min at room temperature. Afterward, 100 μL of L-DOPA solution (0.3% in cacodylate solution) was added. The absorbance was measured every minute for 5 min at 490 nm using a Synergy HT microplate reader (BioTek Instruments, Winooski, VT, USA). Results were calculated using the Beer–Lambert law, using the molar extinction coefficient of L-DOPA (3700). Results were expressed as units of pro-phenoloxidase mL^−1^ of sample.

### 2.5. Gene Expression

#### 2.5.1. Sample Preparation

A total of 5 PL sampled at the end of the experimental trial for molecular biology analysis were homogenized in NZYol (Nzytech, Lisbon, Portugal; *w*/*v* proportion according to the manufacturer’s instructions) using a Precellys 24 tissue homogenizer (Bertin Instruments, Montigny-le-Bretonneux, France).

#### 2.5.2. Gene Expression Analysis

RNA extraction was performed using the NZY total RNA isolation kit (MB13402, NZYTech, Lisbon, Portugal) according to the manufacturer’s instructions. RNA concentration and purity were analyzed by spectrophotometry using DeNovix DS-11 FX (Wilmington, DE, USA). The cDNA was obtained using the NZY first-strand cDNA synthesis kit (MB12501, NZYTech, Lisbon, Portugal). This step was used to standardize the concentration of the samples. Reverse transcription was then performed on a Veriti DX 96-well Thermal Cycler (Applied Biosystems, Foster City, CA, USA). Real-time quantitative PCR (RT-qPCR) was carried out in a CFX384 Touch Real-Time PCR Detection System (Bio-Rad Laboratories, Hercules, CA, USA), using 4.4 μL of diluted cDNA (20 ng µL^−1^) mixed with 5 μL of NZYSpeedy qPCR Green Master Mix^®^ (MB22401, NZYTech, Lisbon, Portugal) and 0.3 μL (10 μM) of each specific primer in a final volume of 10 μL. RT-qPCR was performed in duplicate for each sample. Seven genes were selected and analyzed due to their role in the immune response. Primer efficiency was tested for each gene (Table 3). Cycling conditions were the same between the different genes, consisting of 1 cycle of 95 °C for 10 min, followed by 40 cycles of 2 steps of 95 °C for 15 s and 62 °C for 1 min, with a final cycle at 95 °C for 1 min, followed by 35 s at 62 °C and ending at 95 °C for 0.5 s. The Pfaffl method [22] was used to perform gene expression analyses, and target genes were normalized using *gapdh* as housekeeping [23].

### 2.6. Chemical Analysis

Bromatological analysis of PL carcasses for the determination of their whole-body composition (protein contents for all treatments; lipids and fatty acid composition for FISH and FISHHF) was performed by CCMAR (Portugal) and IRTA (Spain), respectively. For protein determination, *P. vannamei* PL sampled at the end of the trial were freeze-dried, and their total nitrogen (N), hydrogen (H), and carbon (C) content was determined using an elemental analyzer (Elementar Vario EL III, Hanau, Germany). Protein contents were then calculated as *N* × 6.25. For total lipid extraction, samples were homogenized in chloroform/methanol (2:1, *v*/*v*) [24]. Fatty acid methyl esters (FAME) were obtained from total lipids by acid-catalyzed transesterification using 2 mL of 1% H_2_SO_4_ in methanol plus 1 mL of toluene [25]. Afterward, FAME extraction and purification were performed by thin-layer chromatography [26], and separation and quantification were performed by gas–liquid chromatography (Fisons GC8600, Carlo Erba, Milan, Italy) using a 30 m × 0.32 mm capillary column (CP wax 52CB; Chrompack, London, UK) with on-column injection at 50 °C and flame ionization detection at 250 °C. Individual methyl ester identification was performed by comparison to known standards [27].

### 2.7. Data Analysis

Relative growth rate (RGR, % weight day^−1^) was calculated as: RGR = (e.g., 1) × 100, where g = (lnWf − lnWi) × t^−1^. Wf and Wi correspond to the final and initial weights, respectively. FCR was calculated as: FCR = (Fi/Wg), where Fi corresponds to dry feed intake (g) and Wg to the mean wet weight gain (g). Survival was expressed as a percentage and calculated as: S = (Lf/Li) × 100, where Li and Lf correspond to the initial and final number of PL in the tanks, respectively. Differences in growth performance, FCR, survival, oxidative stress biomarkers, energy reserves, immune parameters, and gene expression between dietary treatments were evaluated using one-way ANOVA tests followed by Tukey’s multiple comparison tests. Kruskal–Wallis one-way analysis of variance tests, followed by Wilcoxon pairwise comparison tests, were used when the data did not comply with the one-way ANOVA assumptions. Multivariate linear discriminant analyses (LDA) were performed for gene expression data to evaluate how gene expression contributed to the dissociation of the diets in the discriminant functions generated. A MANOVA was performed to assess discriminatory significance using Wilk’s λ test, after checking that the data complied with the statistical assumptions. The distance between group centroids was measured by Mahalanobis distance, and its significance was inferred by one-way ANOVA tests. Results were expressed as means ± standard deviation (SD). For results expressed as percentages, an arcsine transformation was performed prior to any statistical test: T = ASIN (SQRT (value/100)). The significance level considered was *p* < 0.05 for all tests performed.

## 3. Results

### 3.1. Growth Performance and Survival

At the end of the experiment, no significant differences between treatments were observed in the *P. vannamei* PL mean wet weight and RGR (treatment averages of 255 ± 26 mg and 15.1 ± 0.5% day^−1^, respectively). The FCR values were similar across all dietary treatments, except for FISHHF, which had significantly higher values than KRILL. As for survival, the CTRL and KRILL diets resulted in significantly higher values than FISHHF, while no significant differences between the remaining dietary treatments were observed (Table 4).

### 3.2. Oxidative Stress-Related Biomarkers and Cellular Energy Allocation

**Figure 1:**
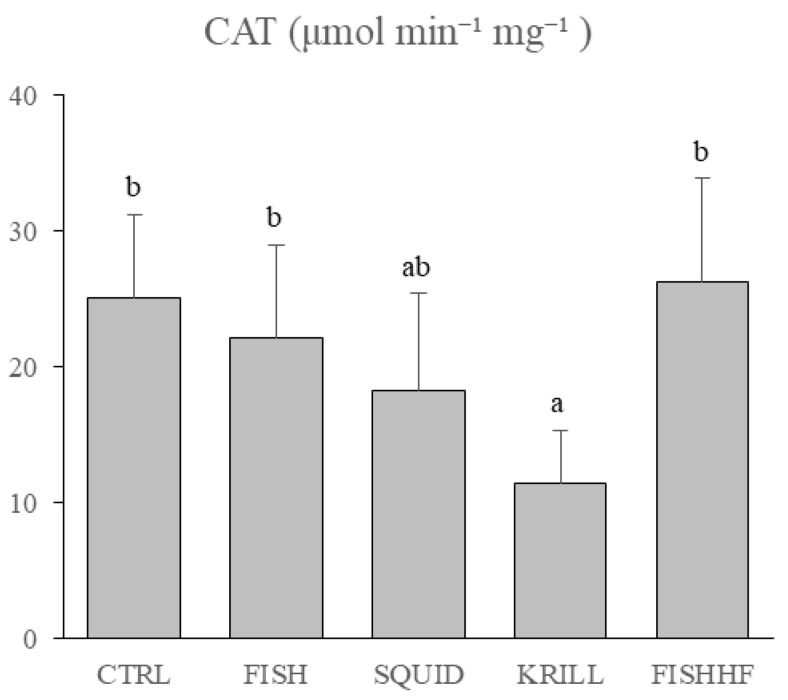
Catalase (CAT) levels (μmol min^−1^ mg^−1^) of whiteleg shrimp (*P. vannamei*) post-larvae (PL) fed the different experimental diets for 21 days. Results expressed as mean ± SD (*n* = 3 experimental units). Different superscript letters indicate statistical differences (*p* < 0.05) between treatments.

**Figure 2:**
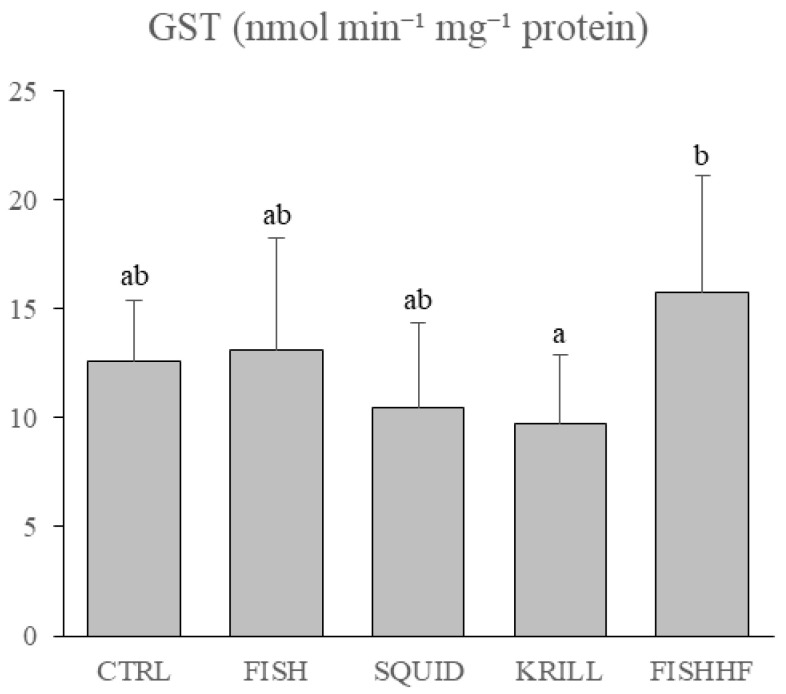
Glutathione S-transferase (GST) levels (nmol min^−1^ mg^−1^ protein) of whiteleg shrimp (*P. vannamei*) post-larvae (PL) fed the different experimental diets for 21 days. Results expressed as mean ± SD (*n* = 3 experimental units). Different superscript letters indicate statistical differences (*p* < 0.05) between treatments.

**Figure 3:**
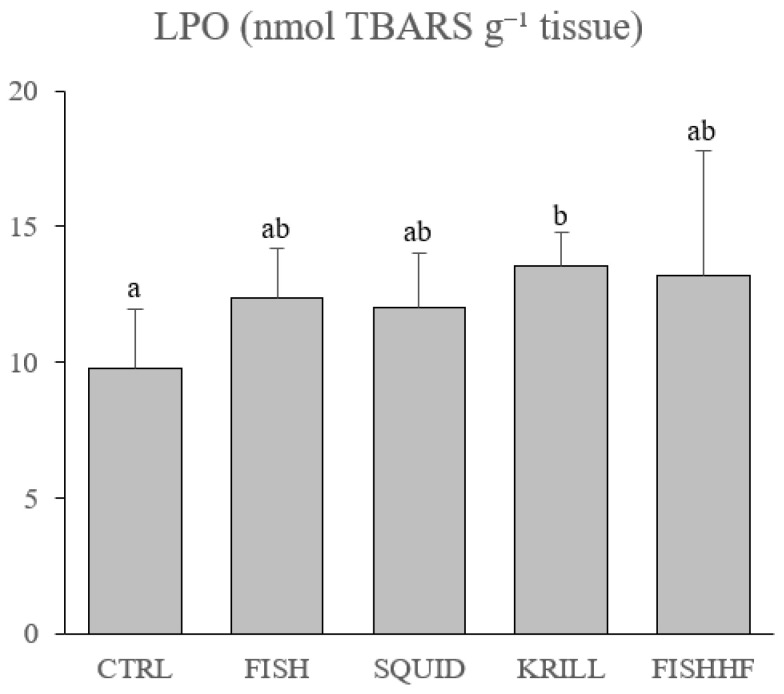
Lipid peroxidation (LPO) levels (nmol TBARS g^−1^ tissue) of whiteleg shrimp (*P. vannamei*) post-larvae (PL) fed the different experimental diets for 21 days. Results expressed as mean ± standard deviation (*n* = 3 experimental units). Different superscript letters indicate statistical differences (*p* < 0.05) between treatments.

Regarding the oxidative stress parameters measured: CAT activity levels were significantly higher in the CTRL and FISHHF diets than in KRILL (Figure 1); GST activity levels were significantly higher in the FISHHF diet than in KRILL, with no significant differences between the remaining treatments (Figure 2); and LPO levels were significantly lower in the CTRL diet than in KRILL, with no significant differences between the remaining treatments (Figure 3). No significant differences between treatments were found in the activity levels of tGSH (15.9 ± 1.1 nmol/min/mg protein, treatment average). As for the energy reserves parameters, no significant differences between treatments were found for lipids (511.4 ± 162.5 mJ/mg tissue, treatment average), carbohydrates (85.7 ± 29.1 mJ/mg tissue, treatment average), and proteins (173.8 ± 71.2 mJ/mg tissue, treatment average) contents, and for EA 770.8 ± 201.8 mJ/mg tissue, treatment average), EC (41.3 ± 13.7 mJ/mg tissue, treatment average) and CEA (20.7 ± 7.6 mJ/mg tissue, treatment average).

### 3.3. Immune Parameters

**Figure 4:**
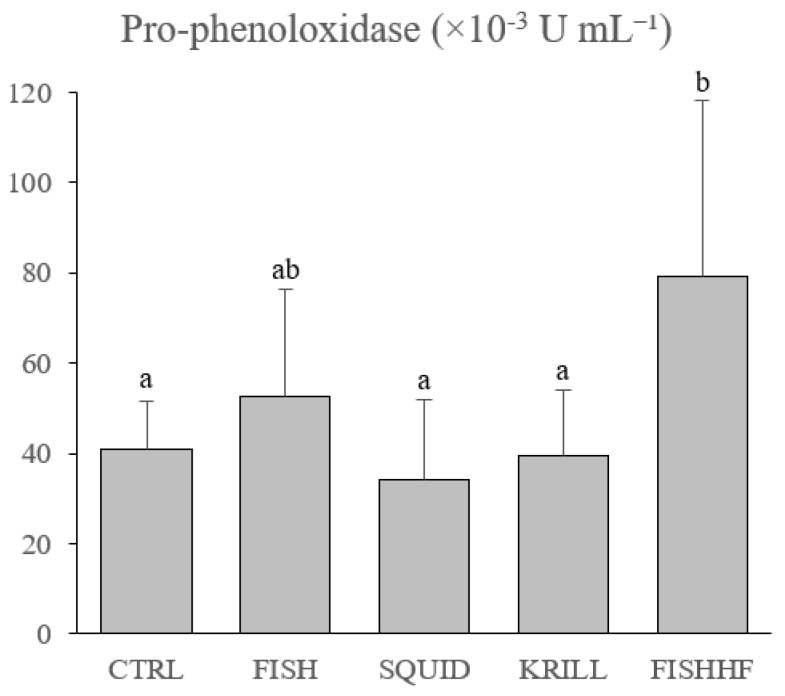
Pro-phenoloxidase levels (×10^−3^ U mL^−1^) of whiteleg shrimp (*P. vannamei*) post-larvae (PL) fed the different experimental diets for 21 days. Results expressed as mean ± standard deviation (*n* = 3 experimental units). Different superscript letters indicate statistical differences (*p* < 0.05) between treatments.

For the immune parameters evaluated, lysozyme activity levels were similar in all treatments (1.27 ± 0.71 µg/mg tissue, treatment average), and pro-phenoloxidase levels were significantly higher in FISHHF than in CTRL, SQUID and KRILL (Figure 4).

### 3.4. Gene Expression Analysis

The normalized relative expression of the *trd* gene was significantly higher in CTRL than in the remaining treatments, excluding KRILL. For the remaining genes, no significant differences between treatments were observed. The ANOVA analysis showed marginally significant differences (*p* = 0.043) between treatments in the normalized relative expression of the *casp* gene. However, no differences between treatments were found in the post hoc Tukey multiple comparison test (Table 5).

**Figure 5:**
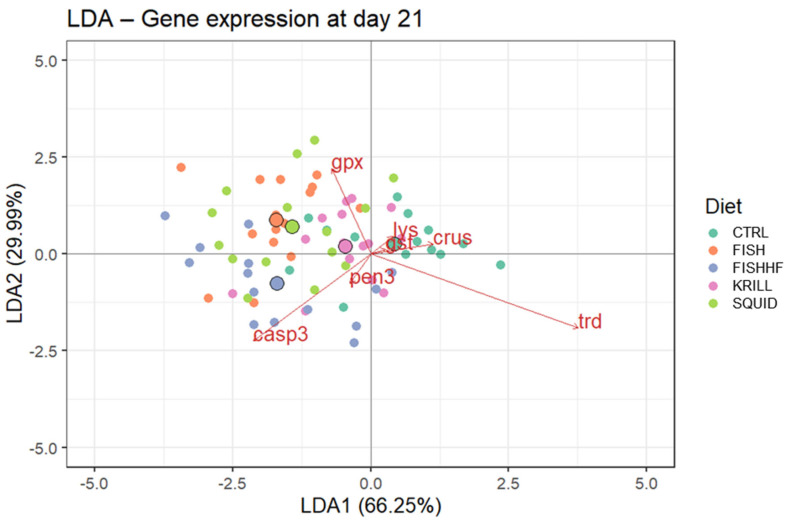
Linear discriminant analysis (LDA) of the relative expression of genes associated with antioxidant defense and immune function (*pen-3*, *crus*, *lys*, *trd*, *gst*, *gpx* and *casp-3*) of whiteleg shrimp (*P. vannamei*) post-larvae (PL) fed the different experimental diets for 21 days. Percentages indicate the main functions (LDA1 and LDA2) discriminant score. Small circles represent diet data distribution while big circles represent diet centroids. Variable loads for both discriminant functions are represented by red arrows. Wilk’s lambda test was significant (*p* < 0.001). In LDA1, CTRL and KRILL were significantly discriminated from FISH and FISHHF, and diet CTRL from SQUID, mainly due to lower mRNA transcripts of *casp-3* and higher transcripts of *trd*. In LDA2, FISHHF was discriminated from FISH and SQUID mainly due to higher mRNA transcripts of the *casp-3* gene.

The LDA of mRNA transcripts of genes associated with antioxidant defense and immune condition analyzed (*pen-3*, *crus*, *lys*, *trd*, *gst*, *gpx*, and *casp-3*) resulted in four discriminant functions, with the first two accounting for 96.24% of the data variability (Wilks λ = 0.425, *p* < 0.001). In the first discriminant function (LDA1, score of 66.25%) (Figure 5), the discriminant analysis revealed a significant group separation between CTRL and KRILL, between FISH and FISHHF, and between CTRL and SQUID. Group discrimination was mainly positively loaded by *trd* and *crus* and negatively loaded by *casp-3*. In the second discriminant function (LDA2, score of 29.99%), a significant separation of FISHHF from FISH and SQUID is also shown (Figure 5). This discrimination was mainly negatively loaded by *casp-3*.

### 3.5. Shrimp Whole Body Composition Analysis

No significant differences between treatments were found in the PL whole-body protein content at the end of the trial (Table 6). The increased lipid levels in the FISHHF diet did not result in higher lipid and total fatty acid contents in the whole-body composition of the PL when compared with PL fed the FISH diet. However, their fatty acid profile differed slightly, with PL fed the FISHHF diet showing significantly higher levels of 16:1 monounsaturated fatty acids; 20:5n-3, 21:5n-3 and total n-3 polyunsaturated fatty acids (PUFA); and significantly lower levels of 18:2n-6 and total n-6 PUFA than PL fed the FISH diet (Table 7).

## 4. Discussion

The RGR values (15.1 ± 0.5% day^−1^; average across treatments) achieved in this trial are in line with those previously described in [1] (15.0 ± 0.3% day^−1^, diet P54 results). However, the PL mean initial weight was slightly higher in this study, which helps explain the higher final weight in the present study. Slightly lower FCR values were also obtained in the current trial (0.9 ± 0.1, average across treatments) than in the previously mentioned study (1.3 ± 0.1, diet P54 results). In a different study, ref. [28] reported slightly higher RGR values (18.6 ± 0.6% day^−1^; average across treatments) and lower FCR values (0.6 ± 0.2; average across treatments), although this trial was conducted with smaller individuals (4.7 ± 0.9 mg initial weight), which may partly explain these differences. As for survival, the CTRL and KRILL treatments obtained values close to 80%, which are only slightly lower than those reported in previous studies [1,28]. Nevertheless, survival values for FISHHF (41.5 ± 15.0%), FISH (45.1 ± 11.7%, marginally with no statistically significant differences from CTRL and KRILL (*p* = 0.077 and *p* = 0.063, respectively), and even those of SQUID (61.6 ± 14.6%) were considerably low, suggesting that these diets may be less suitable than CTRL and KRILL for *P. vannamei* PL at this stage of development and/or for the clear-water RAS husbandry conditions maintained in this study. In fact, the stocking density used in this trial (5000 PL m^−3^) falls within the range typically employed during the nursery phase of *P. vannamei* PL [29], suggesting that diet was the main driver of the observed differences in survival. Still, the survival results observed were superior to those reported by [30,31] when using graded levels of *Schizochytrium* meal as a replacement for fish oil in practical diets for *P. vannamei* PL (40.3–44.5% and 42.7–45.6%, respectively). A different study [32] reported two experiments testing different inclusion levels of protein and lipids in diets for *P. vannamei* PL in clear-water RAS, where survival results were close to 80% in experiment 1 and close to 97% in experiment 2, but weight gain (around 70 and 90 mg in experiments 1 and 2, respectively) and FCR (2.2 and 1.4 in experiments 1 and 2, respectively) were considerably worse than those obtained in the current study. Similar final weight (242 mg) and survival (83%) for *P. vannamei* PL (2.1 mg initial weight) fed a diet containing 51% protein over a 25-day period were described by [33]. Despite the use of different marine protein sources, the absence of growth differences among treatments in this study suggests that all diets provided sufficient nutrients to support growth during the experimental period. Hence, the combined *P. vannamei* PL growth performance and survival results obtained in this trial using diets CTRL and KRILL can be considered very satisfactory and comparable to or superior to those found in similar studies using individuals of the same size and maintained in similar rearing conditions, demonstrating the adequacy of these experimental diets and of the zootechnical conditions implemented. In fact, the inclusion of a blend of fish and squid meals has been previously recommended over using either one alone in diets for juvenile *P. vannamei* reared in clear-water RAS [34]. The same authors reported that krill meal supplementation did not enhance growth and was not included in the diets used in their subsequent study. Accordingly, the combination of fish and squid meals has been designated as the most cost-effective combination of ingredients to be used in feeds for *P. vannamei* [35]. However, recent studies have shown that krill meal inclusion in diets does not negatively affect growth and immunity of juvenile *P. vannamei* [36] and can even improve survival, growth performance, and/or increase feed palatability and intake [5,6,7,8]. Despite the similar growth performance of all dietary treatments in the current trial, these findings corroborate the importance of using either a mixture of fish and squid meals or krill meal as main ingredients in diets for *P. vannamei* PL reared in clear-water RAS to ensure high survival values.

In the present study, the multivariate analysis performed on the relative expression of genes associated with antioxidant defense and immune condition suggests a decline in the health status of PL fed the FISH, SQUID, and FISHHF diets when compared to the CTRL and KRILL dietary treatments (excluding KRILL for SQUID), mainly due to lower whole PL mRNA transcripts of *trd* and *crus,* and higher levels of *casp-3*. The *trd* gene is associated with the redox protein thioredoxin 1, which catalyzes the reduction in disulfide bonds in proteins from different biological systems [37,38]; *crus* encodes PvHm117 crustin P, an antimicrobial peptide involved in innate immunity [39,40]; and *casp-3* gene is related to a protease responsible for programmed cell death and apoptosis in response to the overproduction of reactive oxygen species (ROS) and oxidative stress [41]. Still, PL fed the KRILL diet showed lower activity levels of the antioxidant enzyme CAT. These results may explain the higher LPO levels when compared with CTRL, despite the similar levels of tGSH (free radical scavenger) and GST, a multifunctional enzyme involved in the detoxification of xenobiotics, oxidative defense, and intracellular transport [42], as well as the relative expression of genes associated with antioxidant defense that were evaluated. The lower activity levels of CAT may result in a lower protective capacity against oxidative damage that may occur during these accelerated stages of development, where excessive ROS are likely being produced. Nevertheless, this was not reflected in the survival values, which were similar in both treatments at the end of the trial.

On the other hand, CAT, GST, and pro-phenoloxidase activity levels were significantly higher in the FISHHF dietary treatment than in KRILL. The apparent activation of antioxidant defenses and immune mechanisms in FISHHF may reflect either enhanced physiological preparedness or a compensatory response to increased physiological demands. However, when considered together with the reduced survival, higher FCR, increased pro-phenoloxidase activity, and higher *casp-3* expression observed in this treatment, the results are more consistent with activation of defense pathways in response to physiological stress. The higher mRNA transcripts of *casp-3* further support this interpretation, as this gene is associated with apoptotic processes that can be triggered by oxidative stress and excessive ROS production. Increased ROS generation may also occur during immune activation, potentially explaining the concomitant upregulation of antioxidant defenses [43]. High fish meal diets have been shown to upregulate immunity-related genes, including the pro-phenoloxidase and pro-phenoloxidase activating enzyme genes, in *P. vannamei* when compared to diets containing krill meal as a replacement [5]. Still, in the current study, this was verified only for PL fed the FISHHF diet but not the FISH diet, suggesting that it may have been caused by the increased levels of dietary lipids or PUFAs. However, research on *P. vannamei* PL dietary lipid requirements is limited. The effects of graded dietary lipid levels (9.7–15.5%) on the growth performance, stress tolerance, and immune response of *P. vannamei* PL were evaluated by [44]. The authors reported that the highest lipid levels tested produced the greatest weight gain but also the lowest survival. Levels around 13% upregulated the expression of genes associated with health status and increased PL survival after a hypoxia challenge. Based on these results, the authors indicate 12% to be the optimal dietary lipid level. In the current study, the expression of immune-related genes (*pen-3*, *crus*, and *casp-3*), although not statistically significant, tended to be higher when increasing lipid levels from 14 to 16% in a fish meal-based diet. Moreover, growth performance was not enhanced, and FCR values were higher in PL fed FISHHF than in those fed the KRILL diet. The increase in dietary lipid levels in FISHHF was intended to enhance dietary energy while maintaining protein levels, aiming to promote protein sparing for growth. However, this did not translate into improved growth performance or confer additional physiological benefits. Hence, the results obtained suggest that a lipid level of around 14% seems to be preferable when tailoring diets for *P. vannamei* PL reared in clear-water RAS at this stage of development.

As for the energy reserve parameters assessed, no significant differences were found between treatments in PL protein, lipid, and carbohydrate content, which reflect the similarities in the proximate composition of all diets and in the whole-body composition of the PL from all treatments. No significant differences between treatments were found in the PL whole-body protein and lipid contents at the end of the trial, despite the increased lipid levels in FISHHF. Total fatty acid contents were similar in both treatments, yet the fatty acid profile of PL fed FISH and FISHHF dietary treatments differed slightly. Higher n-3 PUFA and lower n-6 PUFA levels were measured in the whole-body composition of PL fed FISHHF. These results may be associated with the fact that fish oil is rich in n-3 PUFA [10], and the FISHHF diet contained more fish oil than FISH.

One limitation of the present study is that only a single inclusion strategy was evaluated for each marine protein source, and only two dietary lipid levels were tested. In addition, although several physiological and molecular indicators were assessed, the mechanisms underlying the observed differences in survival and physiological responses were not specifically investigated. Future studies should evaluate a broader range of dietary formulations and lipid levels, while incorporating targeted analyses to better elucidate the physiological processes associated with shrimp performance and health.

## 5. Conclusions

In conclusion, this study demonstrates that growth performance alone may not adequately reflect diet suitability in *P. vannamei* post-larvae, as substantial differences in survival and physiological responses were detected despite similar growth outcomes among treatments.

Improved *P. vannamei* PL survival and health status were achieved using diets with a blend of fish and squid meals or krill meal as the main ingredients. Additionally, increasing lipid levels from 14 to 16% in a fish meal-based diet produced similar growth performance but increased FCR and reduced survival. Therefore, these results support the use of combined marine protein sources or krill meal and indicate that lipid inclusion levels not exceeding 14% are preferable when tailoring diets for *P. vannamei* PL reared in clear-water RAS and at this stage of development.

## Figures and Tables

**Table 1:** Composition of the experimental diets for whiteleg shrimp (*P. vannamei*) post-larvae (PL).

% Wet Matter	CTRL	FISH	SQUID	KRILL	FISHHF
Protein	53.5 ± 0.0	54.8 ± 0.0	54.3 ± 0.0	53.5 ± 0.0	54.4 ± 0.0
Lipids	14.5 ± 0.0	14.2 ± 0.0	14.3 ± 0.0	14.3 ± 0.0	16.3 ± 0.0
Ashes	9.6 ± 0.0	12.0 ± 0.0	10.6 ± 0.0	12.0 ± 0.0	12.1 ± 0.0
Phosphorous	1.6 ± 0.0	1.7 ± 0.0	1.6 ± 0.0	1.7 ± 0.0	1.7 ± 0.0
Energy (KJ g^−1^)	19.7 ± 0.0	20.1 ± 0.0	20.2 ± 0.0	20.1 ± 0.0	20.5 ± 0.0

Results expressed as mean ± standard deviation (*n* = 2 experimental units).

**Table 2:** Ingredient list of the experimental diets for whiteleg shrimp (*P. vannamei*) post-larvae (PL).

Ingredients (%)	CTRL	FISH	SQUID	KRILL	FISHHF
Main Protein Source (Squid meal ^1^)	20.0	-	25.0	-	-
Main Protein Source (Fish meal ^2^)	-	42.8	-	-	43.1
Main Protein Source (Krill meal ^3^)	-	-	-	24.7	-
Secondary Marine Protein Sources (Squid meal ^1^, Fishmeal ^2^, Krill meal ^3^, Marine Protein Hydrolysates Mix ^4^)	31.5	9.0	22.5	31.0	9.0
Plant protein Mix ^5^	5.0	16.9	16.9	16.4	16.9
Technological additive Mix ^6^	7.2	4.2	4.2	4.2	4.2
Marine Oils ^7^	3.9	4.5	4.9	4.2	6.4
Plant-based oils ^8^	5.4	3.5	3.8	1.7	3.5
Vitamins and minerals Mix ^9^	6.1	3.0	5.1	4.0	3.0
Starch/Nucleotide sources ^10^	20.9	16.1	17.6	13.8	13.9

^1^, ^2^, ^3^, ^4^ Sopropêche, France; ^5^ F. Duarte, Portugal; ^6^ Azelis, Spain/Weishardt, France/Disproquimica, Portugal; ^7^ Sopropêche, France/Veramaris, The Netherlands; ^8^ Lecico, Germany; ^9^ ADM, Portugal; ^10^ Comfeipas, Portugal/Premix, Portugal.

**Table 3:** Selected genes and specific primers used to evaluate the health status of whiteleg shrimp (*P. vannamei*) post-larvae (PL) at the end of the experimental period.

Gene	Acronym	Efficiency	Annealing Temperature	Accession n°	Amplicon Length (bp)	Primer Sequence (5’-3’)
		(%)	(°C)			
Glyceraldehyde 3-phosphate dehydrogenase	*gapdh*	104.5	60	MG787341.1	166	F: AAAGGTAGGAATTGCCCCCG
						R: AGGGATGAGACTAGCACGACT
PvHm117 crustin P	*crus*	101.4	60	AY488497.1	109	F: GAAACCACCACCAACACCTACTCC
						R: TCTGTGCGGCCTCTTTACGG
Penaeidin-3a	*pen-3*	86.1	60	Y14926.1	137	F: ATACCCAGGCCACCACCCTT
						R: TGACAGCAACGCCCTAACC
Lysozyme C-like	*lys*	73.4	60	XM_027352857	82	F: CGGGAAAGGCTATTCTGCCT
						R: CCAGCACTCTGCCATGTACT
Thioredoxin 1	*trd*	85,3	60	EU499301.1	116	F: TTAACGAGGCTGGAAACA
						R: AACGACATCGCTCATAGA
Glutathione S-transferase	*gst*	99.3	60	AY573381	146	F: AAGATAACGCAGAGCAAGG
						R: TCGTAGGTGACGGTAAAGA
Glutathione peroxidase	*gpx*	86.6	60	XM_027372127.1	117	F: AGGGACTTCCACCAGATG
						R: CAACAACTCCCCTTCGGTA
Caspase 3	*casp-3*	93.8	60	KC660103.1	182	F: ACATTTCTGGGCGGAACACC
						R: GTGACACCCGTGCTTGTACA

**Table 4:** Initial and final weight, relative growth rate (RGR), feed conversion ratio (FCR) and survival of whiteleg shrimp (*P. vannamei*) post-larvae (PL) during the experimental period.

Diet	CTRL	FISH	SQUID	KRILL	FISHHF	*p*-Value
Initial weight (mg)	13.0 ± 2.0					----
Final weight (mg)	225.9 ± 58.8	237.5 ± 48.8	265.7 ± 55.0	281.0 ± 14.0	265.0 ± 57.3	0.657
RGR (% day^−1^)	14.4 ± 1.4	14.8 ± 1.1	15.4 ± 1.1	15.8 ± 0.3	15.3 ± 1.3	0.628
FCR	0.7 ± 0.1 ^b^	1.0 ± 0.1 ^ab^	0.8 ± 0.1 ^ab^	0.6 ± 0.0 ^b^	1.2 ± 0.4 ^a^	0.034
Survival (%)	76.5 ± 9.4 ^b^	45.1 ± 11.7 ^ab^	61.6 ± 14.6 ^ab^	78.0 ± 4.6 ^b^	41.5 ± 15.0 ^a^	0.011

Results expressed as mean ± SD. For initial weight, *n* = 120 observational units; for final weight, FCR, RGR, and survival, *n* = 3 experimental units. Also shown are the *p*-values for a one-way ANOVA test for each parameter. Different superscript letters indicate statistical differences (*p* < 0.05) between treatments in a post hoc Tukey multiple comparison test.

**Table 5:** Relative expression to housekeeping (*gapdh*) of target immune and antioxidant-related genes of whiteleg shrimp (*P. vannamei*) post-larvae (PL) fed the experimental diets for 21 days.

Gene	Acronym	Relative Expression					*p*-Value
		CTRL	FISH	SQUID	KRILL	FISHHF	
Penaeidin-3a	*pen-3*	0.78 ± 0.43	0.70 ± 0.62	0.56 ± 0.48	1.03 ± 0.75	1.26 ± 1.33	0.354
PvHm117 crustin P	*crus*	1.12 ± 0.93	0.34 ± 0.21	0.58 ± 0.52	0.72 ± 0.49	1.02 ± 1.08	0.129
Lysozyme C-like	*lys*	1.00 ± 0.97	0.80 ± 0.54	0.60 ± 0.52	0.88 ± 0.68	0.60 ± 0.35	0.655
Thioredoxin 1	*trd*	1.03 ± 0.44 ^b^	0.41 ± 0.19 ^a^	0.45 ± 0.14 ^a^	0.68 ± 0.24 ^ab^	0.64 ± 0.48 ^a^	<0.001
Glutathione S-transferase	*gst*	1.09 ± 1.00	0.43 ± 0.27	0.56 ± 0.33	0.61 ± 0.54	0.46 ± 0.33	0.224
Glutathione peroxidase	*gpx*	0.71 ± 0.37	0.58 ± 0.31	0.67 ± 0.53	0.54 ± 0.29	0.56 ± 0.52	0.685
Caspase 3	*casp-3*	0.66 ± 0.49	0.71 ± 0.81	0.77 ± 0.56	0.67 ± 0.38	1.55 ± 1.06	0.043

Results expressed as mean ± standard deviation (*n* = 3 experimental units). Also shown are the *p*-values for a one-way ANOVA test for each gene. Different superscript letters indicate statistical differences (*p* < 0.05) between treatments in a post hoc Tukey multiple comparison test.

**Table 6:** Whole body (WBC) protein content of whiteleg shrimp (*P. vannamei*) post-larvae (PL) fed the experimental diets at the end of the experimental period.

Diet	WBC Protein (% DW)	*p*-Value
CTRL	63.4 ± 0.6	0.070
FISH	64.6 ± 1.2	
SQUID	64.8 ± 0.2	
KRILL	64.2 ± 0.6	
FISHHF	64.5 ± 1.7	

Results expressed as mean ± SD (*n* = 3 experimental units). No significant differences between treatments were observed in a one-way ANOVA test.

**Table 7:** Lipid contents and fatty acid composition of whiteleg shrimp (*P. vannamei*) post-larvae (PL) fed the FISH and FISHHF diets at the end of the experimental period.

Shrimp Post-Larvae	Diet		*p*-Value
Whole Body Composition	FISH	FISHHF	
Lipids (%DW)	12.83 ± 0.39	12.75 ± 1.24	----
Fatty acids (mg/g DW)			----
14:0	3.49 ± 0.43	3.26 ± 0.36	----
15:0	1.55 ± 0.20	1.74 ± 0.15	----
16:0	114.77 ± 1.37	112.75 ± 1.62	----
17:0	3.73 ± 0.13	3.67 ± 0.19	----
18:0	51.68 ± 1.25	50.75 ± 1.22	----
20:0	1.81 ± 0.11	1.98 ± 0.17	----
22:0	1.65 ± 0.12	1.58 ± 0.09	----
24:0	1.98 ± 0.37	2.01 ± 0.10	----
Total saturated	180.67 ± 2.05	177.74 ± 2.67	----
16:1	10.79 ± 0.60 ^a^	11.82 ± 0.75 ^b^	0.042
18:1n-9	56.83 ± 4.59	56.79 ± 6.10	----
18:1n-7	32.33 ± 3.92	35.32 ± 1.52	----
20:1	9.58 ± 1.65	8.98 ± 0.87	----
24:1	0.65 ± 0.15	0.64 ± 0.07	----
Total monounsaturated	110.17 ± 6.20	113.55 ± 8.38	----
18:2n-6	89.72 ± 5.28 ^b^	79.30 ± 7.22 ^a^	0.029
18:3n-6	1.22 ± 0.21	1.32 ± 0.41	----
20:4n-6	11.02 ± 0.39	11.77 ± 1.38	----
Total n-6 PUFA	101.97 ± 5.53 ^b^	92.39 ± 5.57 ^a^	0.028
18:3n-3	4.10 ± 0.58	3.48 ± 0.33	----
20:4n-3	0.84 ± 0.19	0.93 ± 0.05	----
20:5n-3	63.30 ± 3.65 ^a^	68.90 ± 3.27 ^b^	0.039
21:5n-3	0.87 ± 0.17 ^a^	1.14 ± 0.17 ^b^	0.038
22:5n-3	4.22 ± 1.01	5.18 ± 0.61	----
22:6n-3	74.29 ± 6.61	77.41 ± 0.61	----
Total n-3 PUFA	147.61 ± 4.53 ^a^	157.04 ± 2.29 ^b^	0.005
Total PUFA	249.58 ± 3.91	249.43 ± 3.67	----
Total FA	555.75 ± 8.55	556.82 ± 9.44	----

Results expressed as mean ± SD (*n* = 3 experimental units). For each parameter, the *p*-values of a one-way ANOVA test are presented when significant. Different superscript letters indicate statistical differences (*p* < 0.05) between treatments in a post hoc Tukey multiple comparison test.

## Data Availability

The data presented in this study are available on request from the corresponding author. The data are not publicly available due to confidentiality issues.
